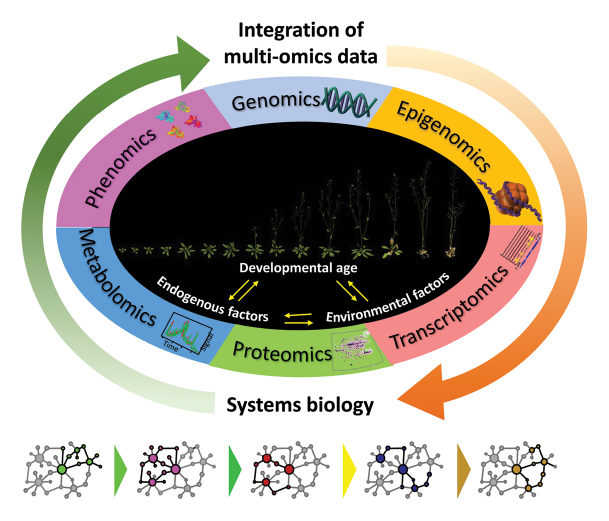# Plant senescence: how plants know when and how to die

**DOI:** 10.1093/jxb/ery011

**Published:** 2018-02-12

**Authors:** Hye Ryun Woo, Céline Masclaux-Daubresse, Pyung Ok Lim

**Affiliations:** 1 Department of New Biology, DGIST, Daegu, Republic of Korea; 2 INRA-AgroParisTech, Institut Jean-Pierre Bourgin, Saclay Plant Sciences, Versailles, France

**Keywords:** Chlorophyll, chloroplast, endogenous signals, exogenous signals, leaf senescence, multi-layered regulatory networks, omics, plant senescence, source–sink interaction

**Virtually all of the cells, tissues and organs in plants age, senesce and eventually die. Senescence is regarded as an evolutionarily acquired process that is critical for plant fitness, and understanding its detailed molecular nature is not only fundamental but also pivotal for the improvement of crop yield and postharvest storage. Impressive progress has been made in revealing new molecular regulatory mechanisms in recent years. In this special issue, reviews span this emerging knowledge – derived from unique biological processes in different types of plant senescence – and highlight key molecular pathways and network-based regulatory mechanisms, as well as their evolutionary implications. The issue also addresses future research perspectives, including new technologies and approaches.**

Plant senescence defines the last stage of a developmental program, which is a degenerative process, but occurs in a temporally coordinated manner (Gan and Amasino, 1997; Lim *et al.*, 2007). It has long been considered as an evolutionarily acquired strategy that is tightly associated with reproduction and survival. During the senescence process, plants integrate multiple internal and external signals as well as information about developmental age through intricate regulatory pathways (Thomas and Stoddart, 1980; Buchanan-Wollaston *et al.*, 2003; Lim *et al.*, 2007). Such integrated characteristics provide the individual with optimal fitness in a given ecological position by regulating the timing of initiation, rate of progression and nature of senescence. Despite its biological importance, however, many molecular mechanisms underlying plant senescence are still largely unknown due to their extreme complexity.

A fundamental question in the field, as yet unanswered, is how plants ‘know’ when and how to die. Six major research themes which address this and constitute the basis of this special issue include (1) changes in the photosynthetic apparatus during leaf senescence, (2) the interaction of endogenous and exogenous signals with senescence pathways, (3) integrative, multi-layered, spatio-temporal networks in leaf senescence, (4) the evolutionary basis of leaf senescence, (5) extended applications of multi-omics technologies to explore genetic elements and their networks that control plant senescence, and integration of multiple types of omics and genetic/physiological data, and (6) source–sink interactions for enhancing crop yield. Although the majority of the reviews focus on leaf senescence, Ma *et al.* (2018) discuss recent progress in understanding hormone-regulated petal senescence; and Patharkar and Walker (2018) address recent advances in understanding the abscission signalling network, as well as future directions for the application of basic abscission research to agriculture.

## Changes in the chloroplasts during plant senescence

During senescence, leaf cells are subject to massive physiological and biochemical changes, including a dramatic metabolic transition from anabolism to catabolism which results in nutrient redistribution to newly developing organs (Lim *et al.*, 2007). The transition from carbon assimilation to nutrient remobilization involves the degradation of cellular structures such as chloroplasts (Masclaux *et al.*, 2000). Two reviews in this issue deal with changes in the chloroplasts, reflecting the rapid progress of research in this area. Chloroplast degradation is co-ordinately regulated by both intra-plastidic and extra-plastidic degradative pathways (Martinez *et al.*, 2008). Otegui (2018) considers the extra-plastidic processes, reviewing how plant cells degrade chloroplast components in vacuoles through autophagy-dependent and -independent trafficking mechanisms. Chlorophyll in chloroplasts is also massively degraded, which results in leaf yellowing, one of the most obvious visible signs of plant senescence (Ougham *et al.*, 2005). Kuai *et al.* (2018) discuss the multi-step, pheophorbide a oxygenase/phyllobilin pathway for chlorophyll breakdown particularly focusing on biochemical and molecular understanding, evolutionary aspects and the transcriptional regulation of the pathway. In addition, Guyer *et al.* (2018) explore the biochemical and structural properties of Arabidopsis pheophytinase (PPH), a key enzyme associated with chlorophyll degradation. The amino acid residues that form the catalytic triad of PPH are also identified through *in silico* modelling of the 3D structure combined with site-directed mutagenesis (Guyer *et al.*, 2018).

## Regulatory mechanisms underlying leaf senescence

The leaf senescence research field has massively enlarged and made great advances over the past couple of decades, but the methods for physiological and molecular investigations are still diverse. In this special issue, Bresson *et al.* (2018) attempt to provide standardized guidelines for leaf senescence analyses in Arabidopsis, including an experimental design for measuring senescence phenotypes at physiological, biochemical and molecular levels.

Recent molecular genetic studies as well as omics analyses of leaf senescence have enormously expanded our knowledge of the underlying molecular mechanisms (Woo *et al.*, 2013; Kim *et al.*, 2016). Kim *et al.* (2018) review recent advances in this area, particularly aspects of dynamic regulation and coordination at multiple levels. Their review also emphasizes the necessity of understanding leaf senescence as a life history strategy from the perspective of system-level spatio-temporal changes and evolution. In addition, recent findings have shed light on the molecular basis of epigenetic and post-transcriptional regulation of leaf senescence (reviewed by Yolcu *et al.*, 2018). As highlighted by Yolcu *et al.* (2018), epigenetic regulation, including chromatin remodelling and modification of DNA, histones and small RNA function, is another key mechanism for modulating gene expression during leaf senescence.

One of the most exciting advances in understanding the regulatory mechanisms of leaf senescence is the identification of diverse transcription factors that play critical roles in the process. Kim *et al.* (2018) highlight the way that dynamic activation and/or deactivation of transcription factors is a key mechanism that controls genome-wide transcriptome changes by continuously integrating developmental and environmental signals. Intriguingly, in this special issue Li *et al.* (2018) address the potential roles of genetic redundancy in senescence-associated transcription factors in the leaf senescence program by analyzing the relationships between gene duplication, gene expression level and phenotypic variation in leaf senescence.

Given that senescence in plants is a highly dynamic process that is precisely coordinated by a complex regulatory network in response to endogenous developmental signals and environmental cues, investigations of leaf senescence should be accomplished by integrative analyses which allow an assessment of the spatio-temporal, dynamic changes that occur in physiological, biochemical and molecular phenotypes. Großkinsky *et al.* (2018) point out the importance of a phenomics approach integrated with multi-omic techniques in senescence research for a better understanding of the process as a complex system.

## Prospects for crop improvement

Leaf senescence is a very important trait for agronomic plants as it limits yield and biomass and modifies nutritional value. For that reason, interest in its manipulation in plant breeding programmes has promoted research projects dedicated to identifying the molecular mechanisms involved in the process, including regulation. The best known biotech application of leaf senescence manipulation technology for plant productivity and quality is the strategy of promoting cytokinin synthesis in senescing leaves by overexpressing *IPT* under the control of senescence-associated promoters (Guo and Gan, 2014). In addition to enhancing grain yield and biomass in many crops, these constructs enhance tolerance to drought stress. In this special issue, Sade *et al.* (2018*b*) show that the down-regulation of *OsCV* expression in rice contributes to water-deficit stress tolerance. *OsCV*, which is involved in chloroplast vesiculation, contributes to chloroplast degradation and is enhanced in ageing leaves and by drought. Silencing *OsCV* contributes to the maintenance of chloroplast integrity and maintains carbon fixation and primary nitrogen assimilation. In their review, Sade *et al.* (2018*a*) show that many transcription factors that play a role in the control of age-induced leaf senescence also play a role in plant stress tolerance. The common feature of age-induced and stress-induced leaf senescence is chloroplast breakdown, and the authors report that many *STAYGREEN* (*SGR*) genes have been shown to play significant roles in regulating chlorophyll breakdown under ‘natural’ and both abiotic and biotic stress-induced senescence. Sade *et al.* (2018*a*) provide a comprehensive overview of the common features regulating source–sink relationships, nutrient recycling and crop performance under age-dependent and stress-induced senescence. They also discuss the role of leaf senescence in perennial plants for different resource management strategies compared with those in annual plants. The review by Yang and Udvardi (2018) focuses on nitrogen management in perennial grasses during leaf senescence, presenting approaches to improve yield, yield stability and nitrogen use efficiency in perennial grasses for forage versus biofuel, including tilling and genome editing. In addition, Großkinsky *et al.* (2018) address the usefulness of a multi-omics approach combined with a phenomics approach in senescence research for agricultural applications. Finally, the metabolomic study presented by Clement *et al.* (2018) shows that leaf senescence programs in leaf veins and leaf lamina tissues of *Brassica napus* present differences that have never been explored before and need a deeper characterization at the molecular level to clarify the nutrient remobilization processes involved in source to sink exchanges.

## Conclusions

Plant senescence is regarded as a complex process in which various environmental signals are integrated into developmental, age-dependent pathways. Thus, mechanisms should exist that sense the age of cells, organs and the whole plant as well as environmental signals, integrate these signals, transduce the signals, select the appropriate pathways and execute the degeneration process. Investigations of the molecular basis underlying these critical steps have been performed and this will continue to be an important area of research. It is more challenging, however, to address how plants coordinate these processes temporally and spatially during senescence. Determining how environmental signals are integrated into information about developmental age will require not only new technologies but also new approaches that are being utilized in the systems biology field (Box 1). This decision process might be better understood through investigating the evolutionary basis of plant senescence. Central questions also remain as to how plants balance environmental signals and developmental signals to allow such a controlled and unique degeneration process. One of the new technological challenges to address these issues would be the establishment of a phenome facility and integration of phenomic data with genomic, proteomic and metabolomic data to interlink the genetic and environmental inputs to plant growth and development with an understanding of the controlling mechanisms from birth to death.

**Box 1. A systems understanding of how a plant knows when and how to die**

Plant senescence constitutes a part of the overall developmental program, in which multiple internal and external signals are integrated into information about developmental age through intricate regulatory pathways. Given the multifaceted nature of the senescence process in plants, integrative analyses which allow an assessment of the dynamic changes that occur in physiological, biochemical and molecular phenotypes is required for a systems understanding of its underlying mechanistic principles. Different multi-omics datasets, such as genomics, epigenomics, transcriptomics, proteomics, metabolomics and phenomics, are integrated for systems biology approaches, and these should provide a more accurate picture of the regulatory networks underlying plant senescence.

**Figure:**